# State of Fragility Fractures Management during the COVID-19 Pandemic

**DOI:** 10.3390/ijerph17217732

**Published:** 2020-10-22

**Authors:** Umberto Tarantino, Ida Cariati, Virginia Tancredi, Donato Casamassima, Eleonora Piccirilli, Riccardo Iundusi, Elena Gasbarra

**Affiliations:** 1 Department of Clinical Sciences and Translational Medicine, Faculty of Medicine and Surgery, “Tor Vergata” University of Rome, Via Montpellier 1, 00133 Rome, Italy; umberto.tarantino@uniroma2.it; 2 Department of Orthopaedics and Traumatology, “Policlinico Tor Vergata” Foundation, Viale Oxford 1, 00133 Rome, Italy; Donatocasamassima87@gmail.com (D.C.); eleonoramed88@gmail.com (E.P.); riccardo.iundusi@uniroma2.it (R.I.); gasbarra@med.uniroma2.it (E.G.); 3 PhD Students’ Program in Medical-Surgical and Biotechnologies and Translational Medicine, Faculty of Medicine and Surgery, “Tor Vergata” University of Rome, Via Montpellier 1, 00133 Rome, Italy; 4 Department of System Medicine, Faculty of Medicine and Surgery, “Tor Vergata” University of Rome, Via Montpellier 1, 00133 Rome, Italy; tancredi@uniroma2.it; 5 Center of Space BioMedicine, Faculty of Medicine and Surgery, “Tor Vergata” University of Rome, Via Montpellier 1, 00133 Rome, Italy

**Keywords:** osteoporosis, COVID-19, fragility fractures, healthcare, aging, pulmonary infection, pandemic

## Abstract

Osteoporosis is a public health concern all over the world. As a chronic condition, it generally requires prolonged medical interventions to limit the risks of further bone loss, impaired skeletal integrity and the onset of fractures. This problem is further complicated by the fact that the abrupt cessation of some therapies may be associated with an increased risk of harm. It is in this context that the COVID-19 pandemic has caused an unprecedented disruption to the provision of healthcare worldwide, exceeding our worst expectations in terms of the number of lives lost and the rapidity at which consolidated economies and healthcare systems are being significantly damaged. In this review, we assessed the challenges and strategies used in the management of osteoporosis and fragility fracture care during the COVID-19 pandemic. We also examined the available evidence and provided clinical recommendations that will require reassessment as the worldwide response to COVID-19 evolves.

## 1. Introduction

The COVID-19 pandemic has undoubtedly had, and will continue to have, a significant impact on the lives of people who live with and are at risk of osteoporosis. It is well known that the clinical expression of COVID-19 consists of massive bilateral interstitial pneumonia, which can lead to severe and life-threatening respiratory failure in a few days [1,2]. Severe interstitial pneumonia has some characteristics, such as pulmonary fibrosis and Chronic Obstructive Pulmonary Disease (COPD), which is a strong independent risk factor for osteoporosis [3]. The etiology of osteoporosis in patients with severe pulmonary disease is complex and variable, and includes chronic systemic inflammation, systemic treatment with corticosteroids, natural variations due to aging and reduced physical performance. In patients affected by COVID-19, such a systemic condition could be associated with a lasting state of hypomobility, which would aggravate the condition of sarcopenia [4] and deplete the skeletal tissue of the mechanical stimuli necessary to maintain its natural trophism. This could lead to a reduction in the patient’s physical capacity, with particular reference to walking, due to clinical conditions and therapeutic ventilation needs.

As a chronic condition, osteoporosis generally requires prolonged medical interventions to limit the risks of further bone loss, impaired skeletal integrity and the onset of fractures. This problem is further complicated by the fact that the abrupt discontinuation of some therapies may be associated with an increased risk of harm [5]. It is in this context that the COVID-19 pandemic has caused an unprecedented disruption to healthcare provision worldwide, exceeding our worst expectations in terms of the number of lives lost and the speed with which well-established economies and healthcare systems have been significantly damaged.

Since to date there is no vaccine or specific drug therapy for COVID-19, the current treatment consists in isolating the patient and managing the clinical symptoms. For this reason, many countries have adopted a series of restrictive measures to mitigate the spread of the virus. However, although these strategies of social distancing have been necessary from a public health perspective, they have understandably introduced challenges in the management of many chronic medical conditions [6], including osteoporosis.

Based on this evidence, the aim of this review was to examine the impact of the COVID-19 pandemic on the management of fragility fractures, with particular reference to diagnosis strategies and changes in medical therapies for osteoporosis.

## 2. Definition of Osteoporosis

Osteoporosis today represents a real health emergency, being a pathology of prevalence and constantly increasing incidence. According to the World Health Organization (WHO), osteoporosis is defined as a systemic skeletal disease characterized by a reduction in bone mass and qualitative skeletal changes [7], which cause an increase in bone fragility and an increased risk of fractures [8].

Osteoporotic fractures occur when mechanical stress applied to the bone exceeds its strength, and result from low energy trauma which normally would not cause a fracture [9]. 

The most frequent fracture sites are the vertebral body, proximal femur, proximal humerus and distal radius [10]. 

The pathogenesis of osteoporosis is multifactorial, and the risk of fractures depends on several independent risk factors, which mainly cause a reduction in Bone Mineral Density (BMD) and “bone quality” (bone geometry, microstructure and turnover, crystalline and organic matrix composition) [11]. Many risk factors act simultaneously through several mechanisms. In general, a low BMD, a medical history of fragility fracture, age, and a family history of osteoporosis are risk factors for an osteoporotic fracture [12]. Osteoporotic fractures of the hip and spine increase the relative risk of mortality; for hip fractures, it is about five to eight times greater in the first 3 months after the event, decreasing over the following 2 years, but remains high at the 10-year follow-up, while the incidence is up to 9% at 1 month after the event, and 36% at 1 year, which is substantially comparable to stroke and breast cancer. Moreover, 50% of patients with hip fracture suffer from a substantial reduction in their level of self-sufficiency which, in approximately 20% of cases, involves long-term institutionalization and longer recovery time [11]. 

## 3. Strategies for the Diagnosis and Orthopedic Management of Fragility Fractures during the COVID-19 Pandemic

The clinical management of patients with fragility fractures is already complex and neglected under normal circumstances. Generally, the diagnosis of osteoporosis is based on the quantitative evaluation of BMD by Dual Energy X-ray Absorbimetry (DEXA) on the femoral neck, which provides the reference site [13]. However, in this pandemic emergency, DEXA services have been suspended, and vulnerable individuals have been recommended to limit their exposure to clinical environments. In these cases, the risk of fracture in patients could be assessed using other clinical prediction tools, such as the Fracture Risk Assessment Tool (FRAX), the Osteoporosis Self-Assessment Tool (OST) and the Khon Kaen Osteoporosis Study score (KKOS) [14,15,16].

Furthermore, it is well known that structured secondary prevention programs, called Fracture Liaison Services (FLS), represent the reference model for the management of adult and older patients after a fragility fracture [17]. The FLS defines a standardized and individualized diagnostic-therapeutic pathway for the management of musculoskeletal complications in patients with a recent fragility fracture, with the aim of reducing the risk of a new fall, the incidence of new fragility fractures and the mortality related to them, as well as ensuring appropriate resource management in fragile patients [18]. However, there is insufficient evidence of the effect on the risk of recurrent fractures. In this regard, a retrospective cohort study was recently conducted to assess whether the implementation of FLS in four Swedish hospitals was associated with a reduced risk of recurrent fractures [19]. In fact, it was observed that simple changes in healthcare routine have the expected effect, resulting in fewer fractures. Therefore, in this pandemic emergency scenario, it would be important to implement FLS, since they have demonstrated their potential clinical and economic efficacy and have been recommended worldwide to reduce the risk of fractures after a first fracture.

Femoral fracture in a frail older patient not affected by COVID-19 is already in itself a very high-risk pathology that requires early and multidisciplinary treatments to reduce the mortality rate, however high. If the patient is also affected by COVID-19 and has pulmonary impairment, the surgical and anesthesiological risks are amplified. In this regard, hospitals have quickly reformulated the care pathways to ensure treatment for infected patients. In particular, orthopedic departments have focused on the traumatology of the elderly, with specific attention to the treatment of femoral fractures in COVID-19 positive patients [20]. The diagnostic and therapeutic pathways to be followed were organized according to a very precise scheme: surgical treatment within 24 h, in an attempt to stabilize the patient, reduction of blood loss, and improvement of respiratory function, with the aim of minimizing the need for admission to the intensive care unit [20]. In this regard, a recent experimental study conducted on 16 older patients with proximal femoral fractures and COVID-19 showed that orthopedic surgery can contribute to overall patient stability, seated mobilization, improvement of physiological ventilation and general patient comfort in bed [20].

## 4. Changes in Medical Therapies for Osteoporosis during the COVID-19 Pandemic

For patients suffering from fragility fractures, pharmacological treatment must be started immediately after the fracture, and multidisciplinary care (orthopaedic surgeon, physiatrist, geriatrician, nurse) is essential. However, considering the current barriers and the ban on going to hospitals and medical offices except in cases of absolute urgency [21], it is possible that there has been an increase in the cases of “underdiagnosis” of fragility fractures, and this may have divided the patients with fractures into two categories, depending on whether or not a diagnosis could be made. Fracture subjects, but in the absence of diagnosis, were extremely penalized in terms of organizing treatment. In the presence of diagnosis, the formulation of the therapy did not always coincide with the patient’s adherence for several reasons, primarily logistical, and this certainly represented a problem, since inadequate adherence to therapy is associated with an increased risk of fractures [22,23].

Continuity of care is not only a prerequisite for successful treatment, but it is also important as regards preserving patient safety on specific treatments [21]. In this regard, since temporary interruptions occurred in the administration of osteoporosis treatments, recommendations for delay or temporary transition to other drug therapies have been provided. However, it should be noted that, during the COVID-19 pandemic, patients already being treated with osteoporosis drugs should continue to receive ongoing therapies, as there is currently no evidence that osteoporosis therapy increases the risk or severity of infection or alters the course of the disease. Furthermore, there is no scientific evidence for impaired fracture healing in patients receiving early treatment for osteoporosis, including bisphosphonates [24]. However, it is well known that the administration of bisphosphonates can cause an inflammatory post-infusion reaction, particularly in naive patients. Symptoms of the inflammatory reaction, including fever and myalgia, can potentially complicate the care of hospitalized patients, triggering a COVID-19 assessment and thus prolonging the hospitalization period [5].

Finally, since the inability to correctly take the drug is a key factor in the management of a bedridden patient and in ventilatory therapy, it may be appropriate to consider reorganizing transient anti-osteoporotic therapies on the basis of the easiest method of administration (subcutaneous, intravenous), with the foresight to remodel the original therapy as soon as the general clinical conditions allow it.

## 5. Recommendations at the Time of COVID-19

**Figure 1:**
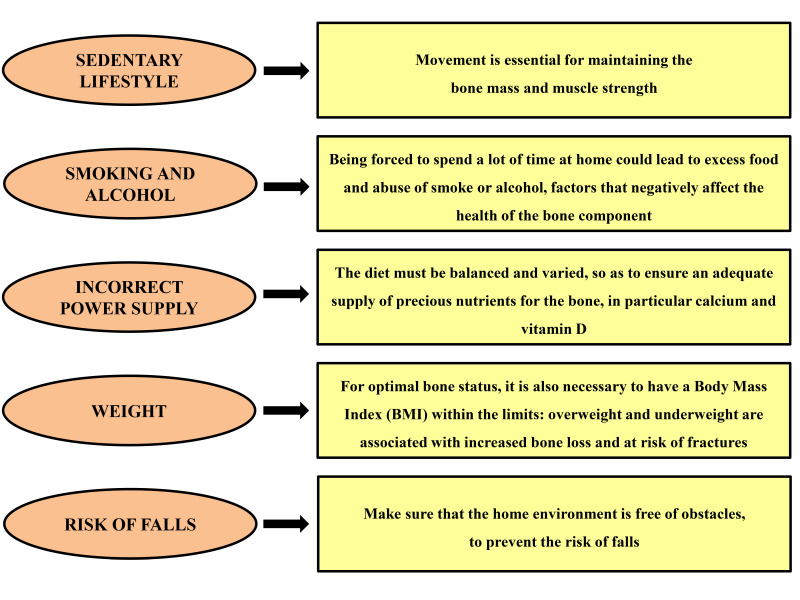
Modifiable factors concerning daily lifestyle.

Due to the COVID-19 emergency, numerous measures have been taken to counter and contain the spread of the infection throughout the national and international territory, such as limitations on outdoor motor and recreational activity, and guided physical and rehabilitation activity in gyms and recovery centers. These restrictive measures have undoubtedly led to a more sedentary lifestyle [25,26]. It is known that physical inactivity and a sedentary lifestyle are among the main risk factors for osteoporosis [5]. Therefore, even if osteoporosis does not increase the risk of contracting COVID-19 or having complications, in order to maintain good skeletal health, it is important to act on the so-called “modifiable” factors (Figure 1), which concern the daily lifestyle. For example, the diet should be balanced and varied to ensure an adequate supply of valuable bone nutrients, particularly calcium and vitamin D. Vitamin D is known to be a fundamental hormone in calcium homeostasis and bone metabolism, and is essential for the development and subsequent maintenance of the skeleton [27,28,29]. It ensures a good level of bone mineralization; in fact, vitamin D deficiency produces serious clinical consequences at the bone level that result in a reduction in bone mass and an increased risk of osteoporotic fractures, in particular of the femur [30,31,32].

Previous observational research has identified vitamin D deficiency as a risk factor for Acute Respiratory Distress Syndrome (ARDS) and ARDS severity [33], which can be a life-threatening complication of COVID-19. There are still no specific data on the role played by vitamin D in coronavirus infection, but patients hospitalized for acute complications by COVID-19 have low vitamin D levels. In any case, the correlation between low vitamin D levels and COVID-19 infection is not immediate, since the patients in question are older subjects in whom vitamin D deficiency is normal if the substance is not administered as a supplement.

## 6. Conclusions

To date, osteoporosis and related fragility fractures, especially in the elderly, represent a real challenge for adequate diagnosis and management during the COVID-19 pandemic. Among the main objectives set, there is that of redesigning the care pathways, giving top priority to the certainty of care, and dedicated paths of access to health facilities and home care. It would be necessary to pay attention to already fractured patients who, to date, for fear of contracting the infection, do not follow adequate care. At the same time, it might be appropriate to consider reorganizing transient anti-osteoporotic therapies based on the easiest method of administration, with the foresight to remodel the original therapy as soon as the general clinical conditions allow it. Given the current emergency, new strategies should be implemented in the management and treatment of osteoporosis to guarantee not only adequate surgical treatment, but also appropriate healthcare after discharge, for example, through the FLS systems with potential clinical and economic efficacy. Furthermore, the importance of ensuring adherence to treatment should be emphasized, as should the implementation of communication with patients and healthcare professionals about the importance of anti-osteoporotic treatment, since continuity of care is a fundamental prerequisite for successful treatment. It is hoped that these recommendations will provide practical and safe guidance for the care and management of patients with osteoporosis during this unprecedented pandemic.
